# Evaluation of the protective roles of alpha-lipoic acid supplementation on nanomaterial-induced toxicity: A meta-analysis of *in vitro* and *in vivo* studies

**DOI:** 10.3389/fnut.2022.991524

**Published:** 2022-09-06

**Authors:** Xiaogang Luo, Dongli Xie, Tong Wu, Wei Xu, Qingyang Meng, Kangli Cao, Jianchen Hu

**Affiliations:** ^1^College of Textile and Clothing Engineering, Soochow University, Suzhou, China; ^2^Shanghai Jing Rui Yang Industrial Co., Ltd, Shanghai, China; ^3^Shanghai Nutri-woods Bio-Technology Co., Ltd, Shanghai, China; ^4^Shanghai Pechoin Daily Chemical Co., Ltd, Shanghai, China; ^5^Shanghai Institute of Spacecraft Equipment, Shanghai, China

**Keywords:** nanomaterials, oxidative stress, inflammation, alpha-lipoic acid, meta-analysis

## Abstract

Extensive exposure to nanomaterials causes oxidative stress and inflammation in various organs and leads to an increased risk of adverse health outcomes; therefore, how to prevent the toxic effects are of great concern to human. Alpha-lipoic acid (ALA) has anti-oxidant and anti-inflammatory activities, suggesting it may be effective to prevent nanomaterial-induced toxicity. However, the results obtained in individual studies remained controversial. We aimed to comprehensively evaluate the effects of ALA supplementation on nanomaterial-induced toxicity by performing a meta-analysis. Databases of PubMed, EMBASE, and Cochrane Library were searched up to May 2022. STATA 15.0 software was used for statistical analysis. Twelve studies were included. Meta-analysis of eight *in vivo* studies showed ALA supplementation could exert significant effects on nanomaterial-induced oxidative stress (by reducing MDA, ROS and increasing GSH, CAT, GPx, and SOD), inflammation (by downregulating NO, IgG, TNF-α, IL-6, and CRP), apoptosis (by activation of pro-apoptotic caspase-3), DNA damage (by a reduction in the tail length) and organ damage (by a decrease in the liver biomarker ALT and increases in brain neuron biomarker AChE and heart biomarker CPK). Pooled analysis of four *in vitro* studies indicated ALA intervention increased cell viability, decreased ROS levels, inhibited cell apoptosis and chelated metal ions. Subgroup analyses revealed changing the levels of GSH, IL-6, and metal ions were the main protective mechanisms of ALA supplementation because they were not changed by any subgroup factors. In conclusion, ALA supplementation may represent a potential strategy for the prevention of the toxicity induced by nanomaterials.

## Introduction

Nanomaterials have been widely utilized in several commercial products, such as electronics, fabrics, drugs, food additive, paint, cosmetics, and sunscreens (1–4). The mass production and consumption of nanomaterials inevitably leads to increased occupational and environmental exposure (5, 6). Nanomaterials can be inhaled, absorbed, ingested, or injected into the body and then transported to various tissues and organs via the bloodstream (7, 8). These studies indicate evaluation of their safety and development of prevention measures are challenges encountered by the scientists.

There have been human, *in vitro* and *in vivo* studies to demonstrate the toxic effects of nanomaterials, with the main mechanisms of inflammatory responses and oxidative damages (9–12). Bello et al. reported exposure to nano-enabled products induced moderate upper airway inflammation and stronger systemic inflammation in healthy operators, evidenced by upregulation of interleukin (IL)-1β, tumor necrosis factor (TNF)-α and interferon-γ (13). The levels of pro-inflammatory leukotrienes type B4, E4 and TNF-α in exhaled breath condensate (EBC) as well as the percentages of chronic bronchitis were higher in nanomaterial workshop employees compared with office employees (14). Multiple regression analysis detected a strongly positive association between occupational exposure in the nano-titanium dioxide (TiO_2_) production facility and EBC level of lipid oxidation marker malonaldehyde (MDA) (15). Nanoparticle (NP) exposure changed the levels of enzymes and molecules involved in oxidative stress [including increased MDA and reduced superoxide dismutase (SOD), glutathione (GSH), glutathione peroxidase (GPx), and catalase (CAT)] (16) and inflammation (TNF-α; IL-6; C-reactive protein, CRP) (17), and cause damages in liver (increased alanine aminotransferase, ALT) (18), kidney (increased creatinine, urea, uric acid) (11), heart (increased creatine phosphokinase, CPK) (17), testes (decreased sperm counts and motility) (19) and brain (decreased AChE activity) (19) in murine models compared with controls. *In vitro* publications showed cell viability was substantially suppressed and cell apoptosis was significantly increased after nanoparticle exposure, which was associated with elevated reactive oxygen species (ROS) (20–22). These findings suggest attenuation of oxidant stress and inflammation may act as potential strategies for preventing the negative effects of nanomaterials.

Recently, nutraceuticals, food or components isolated from food, has been reported to provide health and medical benefits (23). Alpha-lipoic acid (ALA; also known as thioctic acid) is a commonly used nutraceutical that possesses strong anti-oxidant properties because it is an essential cofactor for enzyme complexes involved in mitochondrial oxidative metabolism (24). ALA is also considered to improve inflammatory conditions in the human body (24, 25). Thus, it is hypothesized that supplementation with ALA may represent a cost-effective and safe tool for the prevention of the toxicity induced by nanomaterials. This hypothesis had been demonstrated by some *in vivo* and *in vitro* experiments (17, 21, 26). However, some conflicting results were also identified. Deore et al. did not find significant differences in SOD, GSH, CAT and ROS between ALA + ZnONP- and ZnONP-treated groups (19). Even, Abdelkarem et al. observed that the level of TNF-α was increased in the serum of ZnONP-exposed rats after treatment with ALA (27). Therefore, whether ALA supplementation should be recommended for humans exposed to nanomaterials in the future remains inconclusive.

In the current study, we aimed to perform a comprehensive meta-analysis of all published *in vivo* and *in vitro* studies to examine the effects of ALA supplementation on oxidant and inflammatory markers as well as the consequence of cell viability, death, DNA damage and organ damage. Our results may be useful to guide the clinical use of ALA for human exposed to nanomaterials, particularly occupational workers.

## Materials and methods

### Search strategy

This meta-analysis followed the preferred reporting items for systematic reviews and meta-analysis (PRISMA) 2020 checklist. A systematic search was conducted on electronic databases of PubMed, EMBASE and Cochrane Library up to May 2022 to obtain relevant studies, without language restriction. The search terms included (“nanomaterials” OR “nanoparticle” OR “carbon nanotube” OR “graphene” OR “quantum dot”) AND (“lipoic acid” OR “thioctic acid”). Also, manual checking was done on the reference lists of all relevant studies and previous reviews to identify additional complements.

### Inclusion and exclusion criteria

Studies were included according to the participants, interventions, comparisons, outcomes, and study design (PICOS) criteria: (1) participants (P): animals or cells; (2) intervention (I): the experimental group was co-treated with nanomaterials and ALA; (3) comparison (C): the control group was only administered with nanomaterials; (4) outcomes (O): cell viability, cell death (apoptosis rate, necrosis rate, caspase-3 activity), DNA damage (tail length, tail DNA%), oxidative stress (MDA, ROS, GSH, CAT, GPx, SOD), inflammation (NO, nitric oxide; TNF-α, IL-6, CRP; IgG, immunoglobin G), organ damage (ALT, AChE, CPK, body weight, organ weight) and chelation (concentration of metal ions); and (5) study design (S): controlled trials. The exclusion criteria were: (1) duplications; (2) non-original research (i.e., reviews, case reports, abstracts, letter to the editor, conference proceedings and comments); (3) data could not be available or were reported only in one study; and (4) irrelevant topics. Two authors independently screened the literatures and any disagreements were resolved by consultation with a third reviewer.

### Data extraction

Data extraction was completed by two reviewers independently, including author, publication date, country, cell or animal type, nanomaterial type, nanomaterial dose, ALA dose, ALA administration route, ALA treatment duration, the number of samples in two groups, sample source for analysis of outcomes and related data in two groups (mean ± standard deviation). The data presented only graphically were extracted by using the Engauge Digitizer digitizing software^1^. Any disagreements in data extraction were discussed with another author.

### Quality assessment

The quality of included *in vitro* studies was evaluated by the Toxrtool scale (28, 29), with the values of 0 or 1 point allocated for each item. The Toxrtool scores of the study ranged from 0 to 18 points. Studies with a Toxrtool score of ≥ 11 points were considered as high quality. The quality of each *in vivo* study was evaluated by the SYRCLE risk-of-bias tool based on the following domains: selection, performance, detection, attrition, reporting and other bias (30). Each domain was categorized as ‘low,’ ‘high’ or ‘unclear’ risk of bias if they were labeled as yes, no, unclear to included articles. Quality assessment was performed independently by two reviewers; any disagreements were resolved by a third reviewer.

### Statistical analysis

STATA version 15.0 (Stata Corp., College Station, TX, United States) was used for data analysis. Effect size for the meta-analysis was defined as the standardized mean difference (SMD) and 95% confidence interval (CI). Cochrane’s *Q*-square test and *I*^2^ index were used to measure the between-study heterogeneity. A random-effects model was considered to analyze the pooled effect size for the outcomes when significant heterogeneity was present (*p* < 0.1 and *I*^2^ > 50%); otherwise, a fixed-effects model was utilized. Possible sources of heterogeneity were explored using subgroup analyses based on nanomaterials types, ALA dose, ALA route, ALA duration and sample source for variables with five included datasets. Publication bias was examined by Egger’s linear regression test. The trim-and-fill method was applied to adjust the pooled SMD in the presence of publication bias (*p* < 0.05). Sensitivity analysis with a leave-one-out method was conducted to determine the effect of each study on the overall results.

## Results

### Search results and study selection

**FIGURE 1:**
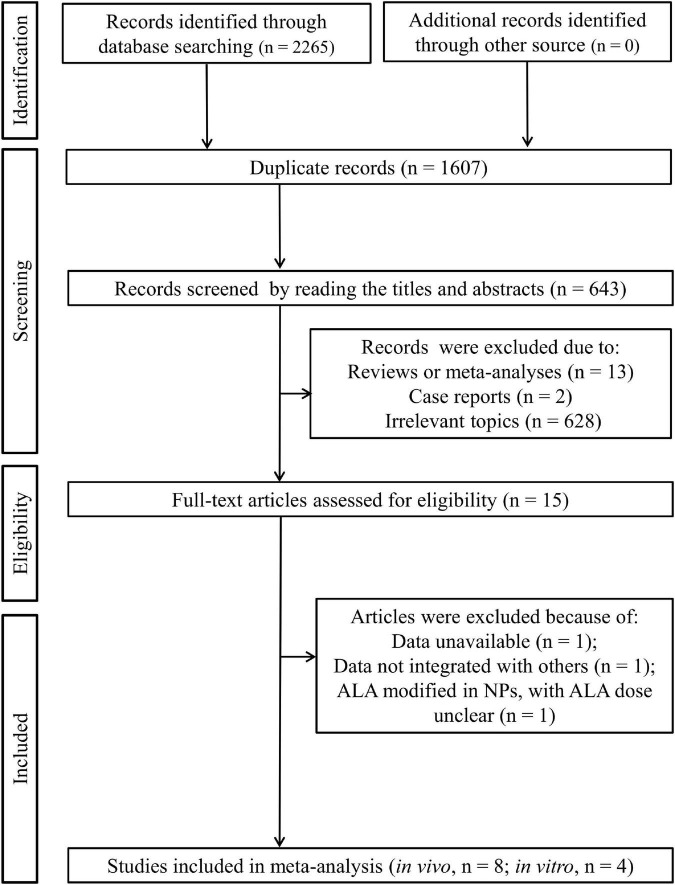
Flow diagram of literature identification according to the PRISMA statement.

**FIGURE 2:**
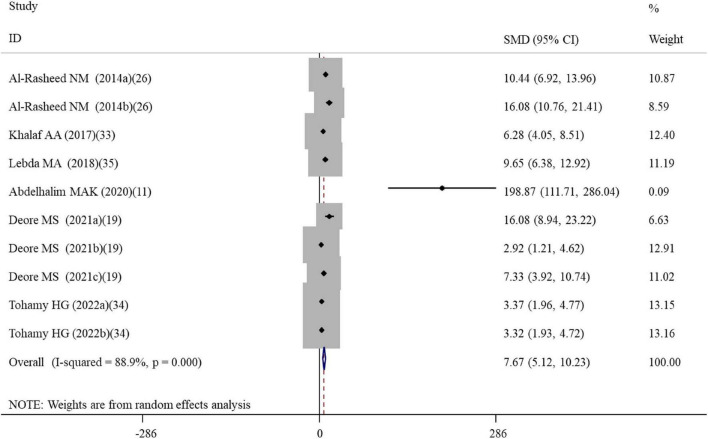
Forest plots assessing the effect of alpha-lipoic acid supplementation on the level of GSH compared with the nanomaterial exposure group. a, b of the study of Al-Rasheed et al. (26) represent treatment with 600 and 1000 mg/kg of ZnONPs; a, b of the study of Tohamy et al. (34) represent the assay of GSH and total GSH; a, b, c of the study of Deore et al. (19) represent the level of GSH in the brain, spleen, and testis tissues. GSH, glutathione; SMD, standardized mean difference; CI, confidence interval.

As shown in Figures 1, 2, 265 published articles were initially identified through systematically searching the electronic databases. After removal of 1,607 duplicates, 658 studies were included for the title and abstract screening. Then, 643 studies were excluded because they were reviews (*n* = 13), case reports (*n* = 2), and irrelevant topics (*n* = 628). The full-texts of the remaining 15 studies were downloaded and read to examine their eligibility. Consequently, three studies were further eliminated because of the following reasons: data unavailable (only the mean provided; *n* = 1) (31); data could not be integrated with other studies (percentage relative controls provided, not specific concentration for outcomes; *n* = 1) (18); ALA modified in NPs and thus only ALA-NP dose was provided, not ALA dose as other studies (*n* = 1) (32). Eventually, eight *in vivo* (11, 17, 19, 26, 27, 33–35) and four *in vitro* (20, 21, 36, 37) studies were included in our meta-analysis.

### Study characteristics

Study characteristics of these 12 eligible articles are shown in Table 1. All *in vivo* studies were performed in the rat model and published from 2013 to 2022; five studies were conducted in Egypt, two in Saudi Arabia and one in the India; four studies investigated the protective roles of ALA for the toxicity induced by zinc oxide nanoparticles (ZnONPs), two for silver nanoparticles (AgNPs), one for copper nanoparticles (CNPs) and gold nanoparticles (GNPs), respectively; the daily doses of ALA were 5, 100, and 200 mg orally or intravenously; the treatment duration ranged from 1 to 8 weeks. *In vitro* studies were published from 2009 to 2021; three studies were conducted in China and one in Canada; each one study was included to explore the protective roles of ALA for the toxicity induced by ZnONPs, AgNPs, cobalt nanoparticles (CoNPs) and quantum dot, respectively. The exposed cells included normal (primary dorsal root ganglia cells, mouse fibroblast cell line Balb/3T3, human pancreatic ductal cell line CRL-4023, human hepatic stellate cells LX-2, human aortic endothelial cells) and malignant types (rat pheochromocytoma cell PC12, human pancreatic ductal adenocarcinoma cell lines BxPc-3, PANC-1, MIA-PaCa2, and BxGEM). The exposed dose of ALA ranged from 50 to 1000 μM and treatment duration ranged from 6 to 24 h.

**TABLE 1:** Characteristics of included articles.

Author	Year	Country	Animals (cells)	No.	Nanomaterial type	Nanomaterial dose	ALA dose	ALA route	ALA duration	Outcomes
Tohamy et al. (34)	2022	Egypt	Rats	20	AgNPs	50 mg/kg	100 mg/kg	Orally	4.28 weeks	MDA, GSH, GPx
Deore et al. (19)	2021	India	Rats	12	ZnONPs	100 mg/kg	5 mg/kg	Orally	2.1 weeks	GSH, CAT, SOD, GPx, ROS, IgG, TNF-α, IL-6, caspase 3 activity, AchE, weight, CPK
Abdelhalim et al. (11)	2020	Saudi Arabia	Rats	12	GNPs	50 μL	200 mg/kg	Intraperitoneally	1 week	MDA, GSH
Lebda et al. (35)	2018	Egypt	Rats	20	AgNPs	50 mg/kg	100 mg/kg	Orally	4.28 weeks	MDA, GSH, CAT, SOD, GPx, AchE
Khalaf et al. (33)	2017	Egypt	Rats	20	CNP	40 mg/kg	100 mg/kg	Orally	8 weeks	ALT, MDA, GSH, CAT, SOD
Abdelkarem et al. (27)	2016	Egypt	Rats	20	ZnONPs	600 mg/kg	200 mg/kg	Orally	3 weeks	NO, IgG, TNF-α, IL-6, CRP, tail length, tail DNA%, calcium
Al-Rasheed et al. (26)	2014	Saudi Arabia	Rats	20	ZnONPs	600,1000 mg/kg	200 mg/kg	Orally	3 weeks	ALT, IgG, TNF-α, IL-6, CRP, GSH, tail length, tail DNA%, caspase 3 activity
Baky et al. (17)	2013	Egypt	Rats	20	ZnONPs	600, 1000 mg/kg	200 mg/kg	Orally	3 weeks	Weight, NO, TNF-α, IL-6, CRP, tail length, tail DNA%, calcium, caspase 3 activity, CPK
An et al. (16)	2021	China	CRL-4023, LX-2, BxPc-3, PANC-1, MIA-PaCa2, BxGEM		AgNPs	1.4 ppm	500, 1000 μM	Co-culture	24 h	Cell viability, ROS, apoptosis
Liu et al. (12)	2020	China	Balb/3T3 cells	6	CoNPs	400 μM	50, 100, 200, 400, and 600 μM	Co-culture	24 h	Cell viability, GSH, ROS, apoptosis, necrosis
Liang et al. (21)	2016	China	HAECs	6	ZnONPs	50 μg/mL	100 μM	Co-culture	6, 12, 24 h	Cell viability, ROS, apoptosis, necrosis
Jain et al. (36)	2009	Canada	PC12, DRG cells	6	Quantum dot	50 μg/ml	200 μM	Co-culture	24 h	Cell viability, GSH

ALA, α-lipoic acid; ZnONPs, zinc oxide nanoparticles; GNPs, gold nanoparticles; CNPs, copper nanoparticle; AgNPs, silver nanoparticles; CoNPs, cobalt nanoparticles; MDA, malonaldehyde; GSH, glutathione; CAT, catalase; SOD, superoxide dismutase; GPx, glutathione peroxidase; ROS, reactive oxygen species; TBARS: thiobarbituric acid reactive substances; NO, nitric oxide; IgG, immunoglobin G; TNF, tumor necrosis factor; IL, interleukin; CRP, C-reactive protein; ALT, alanine aminotransferase; AChE, acetylcholinesterase; CPK, creatine phosphokinase; PC12, rat pheochromocytoma cell; DRG, primary dorsal root ganglia cells; Balb/3T3, mouse fibroblast cell line cells; HAEC, human aortic endothelial cells; CRL-4023, non-malignant human pancreatic ductal cell line; LX-2, human hepatic stellate cells; BxPc-3, PANC-1, and MIA-PaCa2, human pancreatic ductal adenocarcinoma cell lines; BxGEM, gemcitabine-resistant BxPc-3 cells.

### Quality assessment

None of the *in vivo* studies reported sequence generation, allocation concealment, blinding of investigators, random outcome assessment and blinding of outcome assessor; thus, an unclear risk of bias was assigned for them. However, they were at a low risk of bias in terms of baseline characteristics, random housing, incomplete outcome data, selective outcome reporting and others. Therefore, the overall quality of *in vivo* studies was considered to be relatively low. According to the Toxrtool score, all the *in vitro* studies also had high quality scores (Supplementary Table S1).

### Meta-analysis results

Since multiple ALA doses, treatment durations, tissue samples and cell types were designed for some studies, the number of datasets for meta-analysis was larger than the actual number of included articles. The detailed data that were extracted from *in vivo* and *in vitro* studies for each variable are summarized in Supplementary Tables S2, S3, respectively.

#### Effects of alpha-lipoic acid supplementation on oxidative stress in rats exposed to nanomaterials

A total of four, two, ten, five, three, and five datasets examined the effects of ALA supplementation on the levels of oxidative stress-related indicators MDA, ROS, GSH, CAT, GPx, and SOD, respectively (Supplementary Table S2). The pooled analysis using a random-effects model showed that compared with the nanomaterial-exposed group, treatment with ALA induced significant reductions in the levels of pro-oxidant MDA (SMD = –5.53; 95%CI, –8.39 –2.66; *p* < 0.001) and ROS (SMD = –2.84; 95%CI, –5.01 –0.66; *p* = 0.011), while significant increases in the levels of anti-oxidant GSH (SMD = 7.68; 95%CI, 5.12 – 10.23; *p* < 0.001; Figure 2), CAT (SMD = 6.31; 95%CI, 3.61 – 9.00; *p* < 0.001), GPx (SMD = 5.63; 95%CI, 1.70 – 9.55; *p* = 0.005) and SOD (SMD = 4.88; 95%CI, 2.37 – 7.38; *p* < 0.001) (Table 2). The significant beneficial effects of ALA treatment on oxidative stress-related indicators were still present in most of subgroups (except the levels of CAT and SOD in the brain were not improved by ALA supplementation), especially GSH which was not changed by any subgroup variables (Table 3). Although the heterogeneity was still present, it had been decreased by the nanomaterial type and sample source (Table 3), indicating they may be potential sources of heterogeneity.

**TABLE 2:** Meta-analysis with *in vivo* studies to assess effects of ALA for nanomaterial-induced toxicity.

Studies	No.	SMD	95 %CI	*P*_*E*_-value	*I* ^2^	*P*_*H*_-value	Model	Egger p
**Oxidative stress**								
MDA	4	–5.53	−8.39, −2.66	<0.001	87.3	<0.001	R	0.040
ROS	2	–2.84	−5.01, −0.66	0.011	66.6	0.084	R	-
GSH	10	7.68	5.12, 10.23	<0.001	88.9	<0.001	R	<0.001
CAT	5	6.31	3.61, 9.00	<0.001	82.3	<0.001	R	0.016
GPx	3	5.63	1.70, 9.55	0.005	91.7	<0.001	R	0.180
SOD	5	4.88	2.37, 7.38	<0.001	86.9	<0.001	R	0.014
**Inflammation**								
NO	3	–10.49	−17.72, −3.26	0.004	92.0	<0.001	R	0.005
IgG	4	–12.00	−18.07, −5.94	<0.001	91.2	<0.001	R	0.022
TNF-α	7	–5.52	−9.90, −1.13	0.014	95.5	<0.001	R	0.044
IL-6	7	–10.32	−13.76, −6.87	<0.001	85.7	<0.001	R	0.001
CRP	5	–6.28	−9.57, −2.99	<0.001	92.0	<0.001	R	<0.001
**Apoptosis**								
Caspase 3 activity	6	–3.78	−7.19, −0.38	0.029	94.1	<0.001	R	0.392
**DNA damage**								
Tail length	5	–8.00	−12.53, −3.46	0.001	95.6	<0.001	R	0.001
Tail DNA%	5	–1.79	−3.05, −0.53	0.006	85.0	<0.001	R	0.014
**Organ function**								
ALT	3	–6.36	−11.40, −1.32	0.013	93.4	<0.001	R	0.024
AchE	2	6.76	1.12, 13.40	0.019	87.9	0.004	R	-
CPK	3	–10.97	−20.35, −1.60	0.022	96.7	<0.001	R	0.063
Body weight	3	0.33	−0.83, 1.50	0.575	74.4	0.02	R	0.406
Organ weight	4	–1.26	−3.40, 0.88	0.248	90.9	<0.001	R	0.226
Chelation								
Metal content	3	25.62	−6.97, 58.21	0.248	97.3	0.123	R	0.255

ALA, α-lipoic acid; MDA, malonaldehyde; GSH, glutathione; CAT, catalase; SOD, superoxide dismutase; GPx, glutathione peroxidase; ROS, reactive oxygen species; NO, nitric oxide; IgG, immunoglobin G; TNF, tumor necrosis factor; IL, interleukin; CRP, C-reactive protein; ALT, alanine aminotransferase; AChE, acetylcholinesterase; CPK, creatine phosphokinase; SMD, standardized mean difference; CI, confidence interval; F, fixed-effects; R, random-effects; P_H_-value, significance for heterogeneity; P_E_-value, significance for treatment effects.

**TABLE 3:** Subgroup analysis for *in vivo* studies.

Studies	No.	SMD	95% CI	*P*_*E*_-value	*I* ^2^	*P*_*H*_-value	Model
**GSH**							
Nanomaterial type							
CNP	1	6.28	4.05, 8.51	<0.001	−	−	R
GNPs	1	198.87	111.71, 286.04	<0.001	−	−	R
ZnONPs	5	10.02	4.91, 15.13	<0.001	90.2	<0.001	R
AgNPs	3	4.96	2.28, 7.64	<0.001	84.7	<0.001	R
ALA dose							
≤100 mg/kg	7	5.75	3.77, 7.72	<0.001	81.8	<0.001	R
>100 mg/kg	3	17.72	3.79, 31.65	0.013	90.3	<0.001	R
ALAroute							
Orally	9	7.33	5.06, 9.61	<0.001	87.1	<0.001	R
Other	1	198.87	111.71, 286.04	<0.001	−	−	R
ALA duration							
≤2 weeks	4	9.96	1.64, 18.28	0.019	91.5	<0.001	R
>2 weeks	6	7.48	4.65, 10.13	<0.001	89.0	<0.001	R
Sample source							
Kidney	1	198.87	111.71, 286.04	<0.001	−	−	R
Liver	3	10.47	5.33, 15.60	<0.001	84.3	0.002	R
Testis	3	4.01	2.37, 5.66	<0.001	58.8	0.088	R
Brain	2	12.05	5.95, 18.15	<0.001	61.2	0.108	R
Spleen	1	2.92	1.21, 4.62	0.001	−	−	R
**CAT**							
Nanomaterial type							
CNP	1	5.83	3.74, 7.93	<0.001	−	−	F
ZnONPs	3	4.13	2.87, 5.39	<0.001	19.3	0.289	F
AgNPs	1	17.19	11.50, 22.87	<0.001	−	−	F
ALA duration							
≤2 weeks	3	4.19	2.77, 5.62	<0.001	19.3	0.289	F
>2 weeks	2	11.19	0.08, 22.30	0.048	92.6	<0.001	R
Sample source							
Liver	1	5.83	3.74, 7.93	<0.001	−	−	R
Testis	1	3.55	1.62, 5.47	<0.001	−	−	R
Brain	2	10.22	-2.89, 23.33	0.127	94.7	<0.001	R
Spleen	1	6.29	3.31, 9.27	<0.001	−	−	R
**SOD**							
Nanomaterial type							
CNP	1	3.29	1.90, 4.67	<0.001	−	−	R
ZnONPs	3	3.23	1.38, 5.08	0.001	65.6	0.055	R
AgNPs	1	18.57	12.44, 24.70	<0.001	−	−	R
ALA duration							
≤2 weeks	3	3.23	1.38, 5.08	0.001	65.6	0.055	R
>2 weeks	2	10.63	-4.34, 25.60	0.164	95.6	<0.001	R
Sample source							
Liver	1	3.29	1.90, 4.67	<0.001	−	−	R
Testis	1	1.76	0.39, 3.13	0.012	−	−	R
Brain	2	11.46	-1.88, 24.80	0.092	93.9	<0.001	R
Spleen	1	3.60	1.66, 5.54	<0.001	−	−	R
**TNF-α**							
ALA dose							
≤100 mg/kg	2	–4.44	-7.99, -0.89	0.014	76.2	0.041	R
>100 mg/kg	5	–5.92	-12.62, 0.78	0.083	96.9	<0.001	R
ALAduration							
≤2 weeks	2	–4.44	-7.99, -0.89	0.014	76.2	0.041	R
>2 weeks	5	–5.92	-12.62, 0.78	0.083	96.9	<0.001	R
Sample source							
Serum	5	–5.92	-12.62, 0.78	0.083	96.9	<0.001	R
Brain	1	–2.85	–4.53, –1.16	0.001	−	−	R
Spleen	1	–6.50	–9.57, –3.44	<0.001	−	−	R
**IL-6**							
ALA dose							
≤100 mg/kg	2	–6.37	–10.02, –2.71	0.001	61.6	0.107	R
>100 mg/kg	5	–12.08	–17.16, –7.01	<0.001	88.4	<0.001	R
ALAduration							
≤2 weeks	2	–6.37	–10.02, –2.71	0.001	61.6	0.107	R
>2 weeks	5	–12.08	–17.16, –7.01	<0.001	88.4	<0.001	R
Sample source							
Serum	5	–12.08	–17.16, –7.01	<0.001	88.4	<0.001	R
Brain	1	–8.60	–12.53, –4.66	<0.001	−	−	R
Spleen	1	–4.81	–7.20, –2.42	<0.001	−	−	R
**Caspase 3 activity**							
**ALA dose**							
≤100 mg/kg	2	0.41	–8.27, 9.08	0.927	96.6	<0.001	R
>100 mg/kg	4	–5.86	–9.80, –1.92	0.004	93.1	<0.001	R
ALAduration							
≤2 weeks	2	0.41	–8.27, 9.08	0.927	96.6	<0.001	R
>2 weeks	4	–5.86	–9.80, –1.92	0.004	93.1	<0.001	R
Sample source							
Cardiac	2	–4.09	–9.57, 1.38	0.143	94.2	<0.001	R
Liver	2	–7.62	–9.49, –5.75	<0.001	0.0	0.440	F
Brain	1	4.85	2.44, 7.26	<0.001	−	−	R
Spleen	1	–4.00	–6.09, –1.91	<0.001	−	−	R
**Tail length**							
Sample source							
Cardiac	2	–11.40	–14.35, –8.46	<0.001	13.5	0.282	R
Liver	3	–5.40	–9.98, –0.82	0.021	95.3	<0.001	R
**Tail DNA%**							
Sample source							
Cardiac	2	–2.92	–5.91, 0.07	0.056	89.0	0.003	R
Liver	3	–1.15	–2.55, 0.24	0.105	82.8	0.003	R

ALA, α-lipoic acid; NP, nanoparticles; ZnONPs, zinc oxide nanoparticles; GNPs, gold nanoparticles; CNPs, copper nanoparticle; AgNPs, silver nanoparticles; GSH, glutathione; CAT, catalase; SOD, superoxide dismutase; TNF, tumor necrosis factor; IL, interleukin; SMD, standardized mean difference; CI, confidence interval; F, fixed-effects; R, random-effects; P_H_-value, significance for heterogeneity; P_E_-value, significance for treatment effects.

#### Effects of alpha-lipoic acid supplementation on inflammation in rats exposed to nanomaterials

**FIGURE 3:**
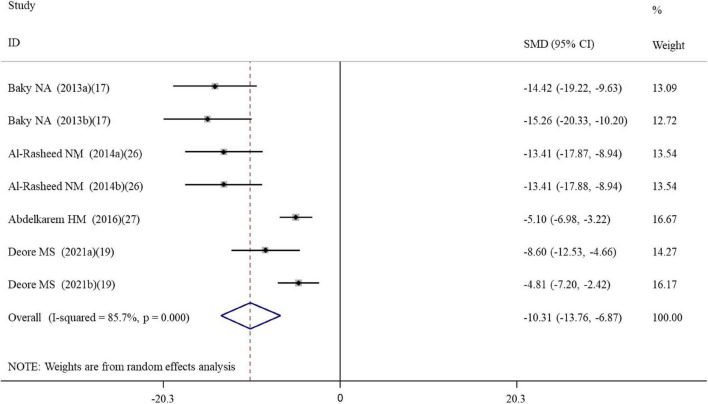
Forest plots assessing the effect of alpha-lipoic acid supplementation on the level of IL-6 compared with the nanomaterial exposure group. a, b of the study of Baky et al. (17) and Al-Rasheed et al. (26) represent treatment with 600 and 1000 mg/kg of ZnONPs; a, b of the study of Deore et al. (19) represent the level of IL-6 in the brain and spleen tissues. IL, interleukin; SMD, standardized mean difference; CI, confidence interval.

Overall, three, four, seven, seven, and five datasets respectively measured the levels of inflammation factors NO, IgG, TNF-α, IL-6 and CRP in nanomaterial-exposed and ALA treatment groups (Supplementary Table S2). The results of meta-analysis using a random-effects model showed that ALA supplementation significantly decreased the levels of NO (SMD = –10.49; 95%CI, –17.72 –3.26; *p* = 0.004), IgG (SMD = –12.00; 95%CI, –18.07 –5.94; *p* < 0.001), TNF-α (SMD = –5.52; 95%CI, –9.90 –1.13; *p* = 0.014), IL-6 (SMD = –10.32; 95%CI, –13.76 –6.87; *p* = 0.014; Figure 3) and CRP (SMD = –6.28; 95%CI, –9.57 –2.99; *p* < 0.001) (Table 2). The inhibiting effect of ALA supplementation on the level of IL-6 was still significant in any subgroups and the heterogeneity was also decreased. The effects of ALA supplementation on the levels of TNF-α seemed to be only significant in the early stage with a low-dose (duration ≤ 2 weeks, *p* = 0.014) (Table 3).

#### Effects of alpha-lipoic acid supplementation on apoptosis in rats exposed to nanomaterials

**FIGURE 4:**
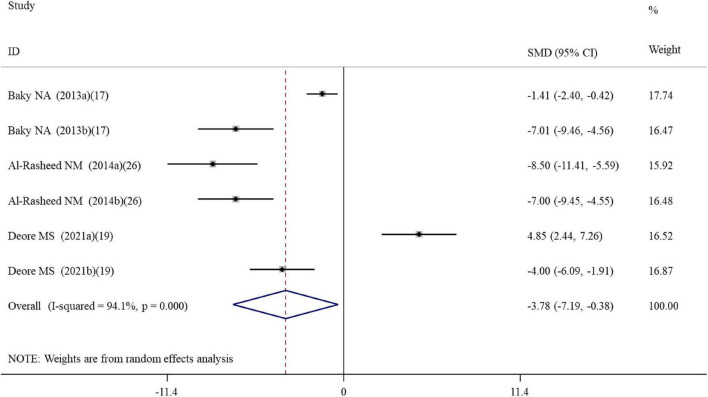
Forest plots assessing the effect of alpha-lipoic acid supplementation on the caspase-3 activity compared with the nanomaterial exposure group. a, b of the study of Al-Rasheed et al. (26) represent treatment with 600 and 1000 mg/kg of ZnONPs; a, b of the study of Deore et al. (19) represent the caspase-3 activity in the brain and spleen tissues. SMD, standardized mean difference; CI, confidence interval.

Six datasets assessed the caspase-3 activity following ALA administration to rats (Supplementary Table S2). Results pooled from the random-effects model showed that compared with the controls, ALA supplementation could remarkably decrease the activity of caspase-3 (SMD = –3.78; 95%CI, –7.19 –0.38; *p* = 0.029) (Table 2 and Figure 4). Subgroup analysis showed that the protective roles of low-dose and short-duration (*p* = 0.927) ALA supplementation on the apoptosis of cardiac tissues (*p* = 0.143) may be limited (Table 3).

#### Effects of alpha-lipoic acid supplementation on DNA damage in rats exposed to nanomaterials

**FIGURE 5:**
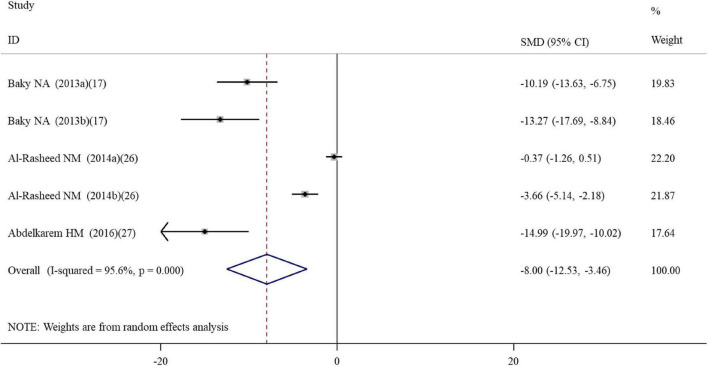
Forest plots assessing the effect of alpha-lipoic acid supplementation on the tail length compared with the nanomaterial exposure group. a, b of the study of Al-Rasheed et al. (26) represent treatment with 600 and 1000 mg/kg of ZnONPs. SMD, standardized mean difference; CI, confidence interval.

Five datasets (Supplementary Table S2) reported the effect of ALA supplementation on DNA damage which used the tail DNA content and the tail length measured by comet assay as metrics. The combined results revealed that ALA consumption resulted in significant decreases in the tail length (SMD = –8.00; 95%CI, –12.53 –3.46; *p* < 0.001; Figure 5) and tail DNA% (SMD = –1.79; 95%CI, –3.05 –0.53; *p* = 0.006) (Table 2). The beneficial effects of ALA treatment on the tail length remained significant after the subgroup analysis based on sample sources (Table 3).

#### Effects of alpha-lipoic acid supplementation on organ function in rats exposed to nanomaterials

Liver function biomarker ALT, brain neuron biomarker AChE and heart function biomarker CPK were analyzed in three, two and three datasets, respectively (Supplementary Table S2). Body weight and organ weight were also recorded in three and four datasets to assess the overall damage (Supplementary Table S2). The pooled results demonstrated that ALA supplementation caused a significant change in the levels of ALT (SMD = –6.36; 95%CI, –11.40 –1.32; *p* = 0.013), AChE (SMD = 6.76; 95%CI, 1.12 – 13.40; *p* = 0.019) and CPK (SMD = –10.97; 95%CI, –20.35 –1.60; *p* = 0.022) (Table 2). Body weight (*p* = 0.575) and organ weight (*p* = 0.248) were not significantly changed by ALA supplementation (Table 2).

#### Effects of alpha-lipoic acid supplementation on metal content in rats exposed to nanomaterials

Metal oxides NP exposure often induced metal ions accumulation in cells or tissues and led to adverse effects. Thus, clearance of metal ions was also an important mechanism to prevent their induced damages. Three datasets detected the concentration of copper, silver and zinc in rats after exposure to CNPs, AgNPs, and ZnONPs as well as the effects of ALA supplementation (Supplementary Table S2). The pooled results showed ALA supplementation did not exhibit an excellent effect of chelating metal ions (SMD = 25.62; 95%CI, –6.97 – 58.21; *p* = 0.248) (Table 2).

#### Effects of alpha-lipoic acid supplementation on the viability of cells exposed to nanomaterials

**FIGURE 6:**
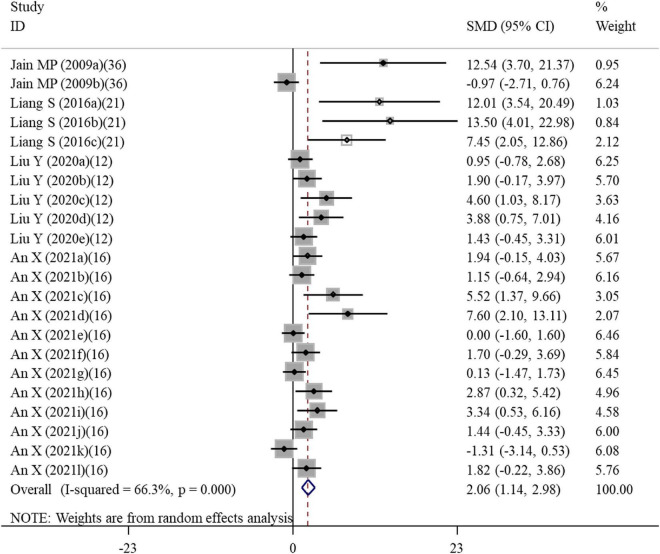
Forest plots assessing the effect of alpha-lipoic acid supplementation on the cell viability compared with the nanomaterial exposure group. a, b of the study of Jain et al. (36) represent the effects on PC12 and DRG cells; a, b, c of the study of Liang et al. (21) represent the effects on HAECs for 6, 12, and 24 h; a, b, c, d, e of the study of Liu et al. (12) represent treatment with 50, 100, 200, 400, and 600 μM alpha-lipoic acid; a, b, c, d, e, f, g, h, I, j, k, l of the study of An et al. (16) represent treatment with 500 and 1000 μM alpha-lipoic acid for CRL-4023, LX-2, BxPc-3, BxGEM, PANC-1, and MIA-PaCa2 cells. SMD, standardized mean difference; CI, confidence interval.

A total of 22 datasets investigated the effects of ALA supplementation on the cell viability (Supplementary Table S3). The pooled results showed that ALA treatment could significantly enhance the viability of cells compared with the nanomaterial exposure group (SMD = 2.06; 95%CI, 1.14 – 2.98; *p* < 0.001) (Table 4 and Figure 6). Except of quantum dot, subgroup analysis further confirmed this significant effect of ALA treatment on the cell viability (Table 5).

**TABLE 4:** Meta-analysis with *in vitro* studies to assess effects of ALA for nanomaterial-induced toxicity.

Studies	No.	SMD	95% CI	*P*_*E*_-value	*I* ^2^	*P*_*H*_-value	Model	Egger *p*
Cell viability	22	2.06	1.14, 2.98	<0.001	66.3	<0.001	R	<0.001
GSH	4	2.46	0.30,5.21	0.081	74.1	0.009	R	0.044
ROS	6	5.45	9.16,1.75	0.004	79.9	<0.001	R	<0.001
Apoptosis rate	11	1.71	3.51,0.10	0.064	78.8	<0.001	R	0.013
Necrosis rate	3	0.73	3.97,5.44	0.760	85.4	0.001	R	0.913
Metal content	8	4.52	6.48,2.56	<0.001	55.2	0.029	R	<0.001

ALA, α-lipoic acid; GSH, glutathione; ROS, reactive oxygen species; SMD, standardized mean difference; CI, confidence interval; F, fixed-effects; R, random-effects; P_H_-value, significance for heterogeneity; P_E_-value, significance for treatment effects.

**TABLE 5:** Subgroup analysis for *in vitro* studies.

Studies	No.	SMD	95% CI	*P*_*E*_-value	*I* ^2^	*P*_*H*_-value	Model
**Cell viability**	22	1.04	0.01, 2.06	<0.001	66.3	<0.001	R
Nanomaterial type							
Quantum dot	2	5.06	−8.10, 18.22	0.451	88.4	0.003	R
CoNPs	5	1.98	0.83, 3.13	0.001	22.1	0.274	F
ZnONPs	3	9.66	5.55, 13.77	<0.001	0.0	0.458	F
AgNPs	12	1.49	0.54, 2.45	0.002	56.6	0.008	R
ALA dose							
≤400 μM	9	4.05	1.68, 6.43	0.001	77.8	<0.001	R
>400 μM	13	1.46	0.59, 2.32	0.001	52.8	0.013	R
ALAduration							
6 h	1	12.01	3.54, 20.49	0.005	-	−	R
12 h	1	13.50	4.01, 22.98	0.005	-	−	R
24 h	20	1.77	0.92, 2.61	<0.001	61.8	<0.001	R
Cell type							
Non-malignant	13	2.88	1.47, 4.29	<0.001	68.7	<0.001	R
Malignant	9	1.27	0.09, 2.44	0.035	62.3	0.007	R
**ROS**	6	–5.45	−9.16, −1.75	0.004	79.9	<0.001	R
Nanomaterial type							
CoNPs	1	–7.45	−12.86, −2.05	0.007	-	−	R
ZnONPs	1	–5.64	−9.86, −1.42	0.009	-	−	R
AgNPs	4	–4.99	−9.74, −0.24	0.039	82.4	0.001	R
ALA dose							
≤400 μM	2	–6.33	−9.65, −3.00	< 0.001	0.0	0.604	F
>400 μM	4	–4.99	−9.74, −0.24	0.039	82.4	0.001	R
ALAduration							
12 h	1	–5.64	−9.86, −1.42	0.009	-	−	R
24 h	5	–5.56	−9.86, −1.25	0.011	81.6	<0.001	R
Cell type							
Non-malignant	4	–5.82	−11.46, −0.17	0.044	83.7	<0.001	R
Malignant	2	–6.51	−15.04, 2.03	0.135	74.6	0.047	R
**Apoptosis rate**	11	–1.71	−3.51, 0.10	0.064	78.8	<0.001	R
Nanomaterial type							
CoNPs	1	–5.23	−9.18, −1.27	0.010	-	−	R
ZnONPs	2	–3.82	−7.98, 0.33	0.071	61.9	0.105	R
AgNPs	8	–0.63	−2.58, 1.33	0.530	77.3	<0.001	R
ALA dose							
≤400 μM	3	–4.03	−6.76, −1.29	0.004	45.2	0.161	F
>400 μM	8	–0.63	−2.58, 1.33	0.530	77.3	<0.001	R
ALAduration							
12 h	1	–2.18	−4.38, 0.20	0.052	-	−	R
24 h	10	–1.72	−3.71, 0.28	0.091	79.7	<0.001	R
Cell type							
Non-malignant	7	–1.79	−3.63, 0.06	0.058	75.1	<0.001	R
Malignant	4	–3.83	−10.09, 2.43	0.230	86.2	<0.001	R
**Metal content**	8	–4.52	−6.48, −2.56	<0.001	55.2	0.029	R
Nanomaterial type							
Quantum dot	2	–5.59	−8.56, −2.62	<0.001	0.0	0.809	F
CoNPs	2	–10.73	−16.11, −5.35	<0.001	0.0	0.974	F
AgNPs	4	–2.55	−3.83, −1.26	<0.001	35.6	0.198	F
ALA dose							
≤400 μM	4	–6.79	−9.39, −4.19	<0.001	0.0	0.432	F
>400 μM	4	–2.55	−3.83, −1.26	<0.001	35.6	0.198	F
Cell type							
Non-malignant	5	–4.47	−7.37, −1.57	0.003	67.4	0.015	R
Malignant	3	–5.15	−7.41, −2.88	<0.001	0.0	0.902	F

ALA, α-lipoic acid; NP, nanoparticles; ZnONPs, zinc oxide nanoparticles; AgNPs, silver nanoparticles; CoNPs, cobalt nanoparticles; ROS, reactive oxygen species; SMD, standardized mean difference; CI, confidence interval; F, fixed-effects; R, random-effects; P_H_-value, significance for heterogeneity; P_E_-value, significance for treatment effects.

#### Effects of alpha-lipoic acid supplementation on oxidative stress of cells exposed to nanomaterials

Four and six studies assessed the effects of ALA supplementation on the level of GSH and ROS in cells exposed to nanomaterials, respectively (Supplementary Table S3). The pooled results showed that the level of GSH in cells was only increased by ALA supplementation at a marginal significance (*p* = 0.081), while the level of ROS was statistically decreased by ALA (SMD = –5.45; 95%CI, –9.16 –1.75; *p* = 0.004) (Table 4). Subgroup analysis further demonstrated the protective effects of ALA treatment on ROS, particularly for non-malignant cells (Table 5).

#### Effects of alpha-lipoic acid supplementation on death of cells exposed to nanomaterials

Apoptosis and necrosis rates determined by flow cytometry were recorded in eleven and three datasets, respectively (Supplementary Table S3). As shown in Table 4, the overall SMD of ALA treatment compared with the control was –1.71 (95% CI: –3.51 to 0.1) for the apoptosis rate and 0.73 (95% CI: –3.97 to 5.44) for the necrosis rate, indicative of no statistical difference between these two groups. However, a significant inhibitory effect of ALA treatment on the cell apoptosis was observed in the subgroup with a low dose (≤400 μM; SMD = –4.03; 95%CI, –6.76 –1.29; *p* = 0.004) under a fixed-effect model (Table 5), implying supplementation with low-dose ALA may be effective to prevent the apoptosis of cells exposed to nanomaterials.

#### Effects of alpha-lipoic acid supplementation on metal content in cells exposed to nanomaterials

Eight datasets detected the concentration of cadmium, cobalt and silver in cells after exposure to cadmium-quantum dot, CoNPs and AgNPs as well as the effects of ALA supplementation (Supplementary Table S3). The pooled results showed that ALA supplementation significantly cleared the metal ions caused by nanomaterials (SMD = –4.52; 95%CI, –6.48 –2.56; *p* < 0.001) (Table 4). This chelating effect was still significant in all subgroup analyses (Table 5).

### Publication bias and sensitivity analysis

**FIGURE 7:**
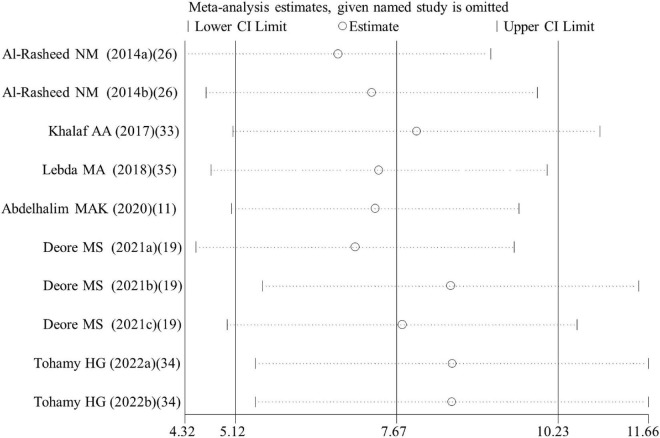
Sensitivity analysis for GSH. GSH, glutathione; CI, confidence interval.

Egger’s test showed no evidence of publication bias for analysis of GPx (*p* = 0.180), caspase 3 activity (*p* = 0.392), CPK (*p* = 0.063), body weight (*p* = 0.406), organ weight (*p* = 0.226), and necrosis rate (*p* = 0.913), while publication bias existed for analysis of MDA (*p* = 0.04), rat GSH (*p* < 0.001), CAT (*p* = 0.016), SOD (*p* = 0.014), NO (*p* = 0.005), IgG (*p* = 0.022), TNF-α (*p* = 0.044), IL-6 (*p* = 0.001), CRP (*p* < 0.001), tail length (*p* = 0.001), tail DNA% (*p* = 0.014), ALT (*p* = 0.024), cell viability (*p* < 0.001), cell GSH (*p* = 0.044), cell ROS (*p* < 0.001), and apoptosis rate (*p* = 0.013). Therefore, the trim and fill method was used to adjust the pooled SMD for these variables with publication bias. The results showed the SMD for MDA, NO, IgG, TNF-α, IL-6, CRP, tail length, tail DNA%, ALT, ROS and apoptosis was the same as the results before correction. Although SMD was decreased, a statistical difference was still present for rat GSH (SMD = 4.15; 95%CI, 1.37 – 6.92; *p* = 0.003), CAT (SMD = 4.36; 95%CI, 1.44 – 7.27; *p* = 0.003), SOD (SMD = 2.80; 95%CI, 0.07 – 5.53; *p* = 0.044) and cell viability (SMD = 1.04; 95%CI, 0.01 – 2.06; *p* = 0.047). The level of GSH in cells was still demonstrated not to be significantly changed by ALA after correction (SMD = 0.98; 95%CI, –1.73 – 3.69; *p* = 0.477). The leave-one-out sensitivity analysis indicated that removing each of the dataset had no significant effect on the pooled effect size (Figure 7).

## Discussion

Due to the anti-oxidant and anti-inflammatory effects of ALA, oral supplementation of ALA has been recommended in clinic for the prevention and treatment of several oxidant- and inflammatory-related diseases (i.e., diabetes, metabolic syndrome, cardiovascular disease, rheumatoid arthritis, et al.) and meta-analysis of randomized clinical trials also confirmed its beneficial effects (25, 38–41). However, only *in vitro* and *in vivo* studies focused on the roles of ALA supplementation on nanomaterial-induced oxidant and inflammatory injuries currently and controversial results were reported. Therefore, our study, for the first time, attempted to comprehensively evaluate the effects of ALA for cells and animals exposed to nanomaterials by performing a meta-analysis, which may provide supporting evidence for the clinical use in the future. As a result, our meta-analysis of eight *in vivo* studies showed that ALA administration could exert significant effects on nanomaterial-induced oxidative stress (manifested by reducing MDA, ROS and increasing GSH, CAT, GPx and SOD), inflammation (evidenced by downregulating NO, IgG, TNF-α, IL-6, and CRP), apoptosis (manifested by activation of pro-apoptotic caspase-3), DNA damage (represented by a reduction in the tail length) and organ damage (represented by a decrease in the liver biomarker ALT and increases in brain biomarker AChE and heart biomarker CPK). Pooled analysis of four *in vitro* studies also indicated ALA intervention increased cell viability, decreased ROS levels and inhibited cell apoptosis. These results confirmed the protective roles of ALA for nanomaterial-induced toxicity by reduction of both inflammatory and oxidative mediators, which were in line with previous meta-analysis studies (25, 39, 40, 42).

It is evident that the heterogeneity was present for analysis of several variables; thus, subgroup analyses were performed to explore the influence of various factors. The results of *in vivo* studies showed that the beneficial effects of ALA supplementation on the levels of GSH and IL-6 were not changed by any subgroup factors, suggesting improvement on these two variables may be the main mechanisms of ALA supplementation for nanomaterial-induced toxicity. ALA-induced increase of the GSH content may be, on one hand, attributed to its ability to supply the cysteine precursor of GSH by metabolic reduction of lipoic acid to dihydrolipoic acid; on the other hand, ALA could promote the regeneration of GSH by activating nuclear factor erythroid 2-related factor 2 (Nrf2)/antioxidant responsive element signaling pathway (43–45) and then upregulating the Nrf2-downstream genes γ-glutamate-cysteine ligase (46) and glutathione reductase (34), both of which are key enzymes for GSH synthesis. The beneficial effect of ALA against IL-6 was associated with the increased GSH concentrations (47, 48). ALA was also reported to enhance the Nrf2/heme oxygenase 1 pathway signaling and inhibit the activation of nuclear factor-kappa B (NF-κB), ultimately markedly suppressing the transcription of NF-κB downstream genes pro-inflammatory cytokine IL-6 (49, 50). The subgroup analysis of TNF-α showed that ALA supplementation exerted a significant inhibition effect on the level of TNF-α at the early stage (duration ≤ 2 weeks). This result may be associated with the fact that TNF-α is located upstream of the cytokine cascade (51). Therefore, at the late stage, the level of TNF-α may be similar in two groups, while downstream cytokine IL-6 was abundantly produced and thus, the effect may be more obvious for IL-6.

In addition to anti-oxidant and anti-inflammatory effects, ALA was considered as a metal chelator to prevent metal ion-induced toxicity (19, 20, 33, 35, 37). Hereby, in this study, we also analyzed the metal ions of NPs in animals and cells. The meta-analysis of *in vitro* studies (*n* = 8) demonstrated the significant metal-chelating capacity of ALA, while no statistical effects were observed for analysis of *in vivo* studies (*n* = 3). This negative metal detoxification effect indicated by *in vivo* studies may result from a meta-analysis with a small sample size. Furthermore, the different binding affinities of ALA to different ions may also explain this difference between *in vitro* and *in vivo* analysis results (52).

Clinically, ALA is commonly used for the treatment of diseases related to the nervous system (53, 54). Although the neuroprotective mechanisms may be complex, anti-oxidant and anti-inflammatory effects of ALA play main roles (53–57), indicating ALA supplementation may antagonize nanomaterial-induced neurotoxicity. In line with the expectation, we found the AchE, an enzyme that mediates the neuromuscular impulse transmission and promotes regeneration of neurites, in rats exposed to nanomaterials was significantly increased by ALA treatment, which was accompanied by improved pathological changes of cerebrum neuron (19, 35). Autonomic neuropathy causes a wide range of cardiac disorders and thus ALA therapy was also attempted for patients with cardiac dysfunction (58, 59) and animal models (60, 61), with the results of improving cardiac functions through suppressing oxidative and inflammatory mediators. Similar to these findings, we also found ALA had a protective role for nanomaterial-induced cardiotoxicity, with CPK significantly decreased (17). Liver tissue is the primary site for detoxification and therefore liver damage is one of the most common consequences after exposure to the toxins, including nanomaterials (33). Prevention of hepatotoxicity was an important goal for the use of ALA. As anticipated, serum ALT (a liver biomarker) enzyme activity in rats that received nanomaterials was significantly reduced after administration of ALA, with the mechanisms associated with an improvement of oxidative and inflammatory status (26, 33).

Oxidative and inflammatory mediators contribute to the ultimate damages in organs since they could react with DNA molecule to cause single-, double-strand breaks and alkali-labile sites (62, 63) and DNA damage could subsequently trigger cell apoptosis via regulating signal transducers including CHK2, p53, E2F-1 and caspases (64). During comet assay, DNA fragmentation migrates into the electric field and forms the comet’s tail; hereby, the tail length reflects the degree of DNA damage. Reduction of the tail length and cell apoptosis (caspase-3 activity) in our results may illustrate the beneficial effects of ALA administration for nanomaterial-induced toxicity.

The present meta-analysis has some limitations. First is the limited number of included *in vivo* and *in vitro* studies, which may affect the pooled effect size for some indicators (such as GSH in cells and metal ion content in rats) and result in the effects on other tissue (i.e., lung, kidney, testes) damages that could not be evaluated. Second, the eligible studies were heterogeneous, and the between-study heterogeneity could not be eliminated by the subgroup analysis, which made our conclusion be interpreted with great caution. Third, included animal studies were at an unclear risk of bias during the quality assessment of domains involving the randomization, allocation concealment, and blinding of outcome assessment. Fourth, the majority of data were extracted from the bar graphs, which may lead to some differences from the real data. Fifth, ALA exists in the form of two different optical isomers [(+) or (-) enantiomers]. There was evidence that (+)-ALA was more active than (±)- or (-)-enantiomers in reducing oxidative stress and may be more effective against nanomaterial-induced injuries (55, 56). However, no included studies reported the isomer information of used ALA. Sixth, although our subgroup analyses suggested the ALA dose seemed not to influence the anti-oxidant, anti-inflammatory and chelation effects, it was known that ALA was also not completely free from side effects, particularly (–) enantiomer-treated animals that were found to display more marked organ toxicity signs (57). Thus, low-dose ALA should be recommended theoretically. However, a limitation was that the dose varied even if they were under the range set by us (≤100 mg/kg in animals or ≤400 μM in cells). Based on these limitations, we consider more experiments are needed to confirm the protective effects of ALA (with specific dose, isomer, duration, et al.) on nanomaterial-induced toxicity before it is recommended for clinical application. We also consider a bibliometric analysis by searching the Web of Science with more broad keywords and using Bibliometrix, VOSviewer, or CiteSpace software to find out the future research hotspot, such as other nutritional components which may be suitable to combine with ALA to lower ALA dose and ALA-brought toxicity.

## Conclusion

Findings of the current meta-analysis suggest that consumption of ALA may provide the beneficial effects on reducing the inflammatory mediator IL-6, increasing the anti-oxidant GSH and chelating metal ions, which ultimately prevents the cell death, DNA and organ damages induced by nanomaterials.

## Data availability statement

The original contributions presented in the study are included in this article/Supplementary material, further inquiries can be directed to the corresponding author/s.

## Author contributions

XL and JH conceived the idea and designed the study. XL and DX collected the data, performed the statistical analysis, and wrote the manuscript. TW, WX, QM, and KC contributed to the interpretation of the results. JH revised the manuscript. All authors approved the final version of the manuscript.

## Footnotes

1
http://digitizer.sourceforge.net/
