# Supplementary material for: Evaluation of the protective roles of alpha-lipoic acid supplementation on nanomaterial-induced toxicity: A meta-analysis of in vitro and in vivo studies
Source: Front Nutr. 2022 Sep 6;9:991524. doi: 10.3389/fnut.2022.991524 (PMC9486203; doi:10.3389/fnut.2022.991524)
Supplement: Supplementary file 1 [file Table_1.DOCX]

**Table S1 Quality assessments**

|  | Toxrtool checklist of study quality | | | | | | | | | | | | | | | | | | | |
| --- | --- | --- | --- | --- | --- | --- | --- | --- | --- | --- | --- | --- | --- | --- | --- | --- | --- | --- | --- | --- |
| Reference  (in vitro) | (1) | (2) | (3) | (4) | (5) | (6) | (7) | (8) | (9) | (10) | (11) | (12) | (13) | (14) | (15) | (16) | (17) | (18) | Total | Reliability of evidence |
| An X | 1 | 1 | 1 | 0 | 1 | 1 | 1 | 1 | 1 | 1 | 1 | 1 | 1 | 1 | 1 | 1 | 1 | 1 | 17 | Reliable without  restrictions |
| Liu Y | 1 | 0 | 1 | 0 | 1 | 1 | 1 | 1 | 1 | 1 | 1 | 1 | 1 | 1 | 1 | 1 | 1 | 1 | 16 | Reliable without  restrictions |
| Liang S | 1 | 0 | 1 | 0 | 1 | 1 | 1 | 1 | 1 | 1 | 1 | 1 | 1 | 1 | 1 | 1 | 1 | 1 | 16 | Reliable without  restrictions |
| Jain MP | 1 | 0 | 1 | 0 | 1 | 1 | 1 | 1 | 1 | 1 | 1 | 1 | 1 | 1 | 1 | 1 | 1 | 1 | 16 | Reliable without  restrictions |
|  | SYRCLE checklist of study quality | | | | | | | | | | | | | | | | | | | |
| Reference  (*in vivo*) | Selection bias | | | | | | Performance bias | | | | Detection bias | | | | Attrition bias | | Reporting bias | | Other | |
|  | SG | | BC | | AC | | RH | | BI | | ROA | | BOA | | IOD | | SOR | |  |  |
| Tohamy HG | Unclear | | Low | | Unclear | | Low | | Unclear | | Unclear | | Unclear | | Low | | Low | | Low | |
| Deore MS | Unclear | | Low | | Unclear | | Low | | Unclear | | Unclear | | Unclear | | Low | | Low | | Low | |
| Abdelhalim MAK | Unclear | | Low | | Unclear | | Low | | Unclear | | Unclear | | Unclear | | Low | | Low | | Low | |
| Lebda MA | Unclear | | Low | | Unclear | | Low | | Unclear | | Unclear | | Unclear | | Low | | Low | | Low | |
| Khalaf AA | Unclear | | Low | | Unclear | | Low | | Unclear | | Low | | Unclear | | Low | | Low | | Low | |
| Abdelkarem HM | Unclear | | Low | | Unclear | | Low | | Unclear | | Unclear | | Unclear | | Low | | Low | | Low | |
| AL-RASHEED NM | Unclear | | Low | | Unclear | | Low | | Unclear | | Unclear | | Unclear | | Low | | Low | | Low | |
| Baky NA | Unclear | | Low | | Unclear | | Low | | Unclear | | Unclear | | Unclear | | Low | | Low | | Low | |

(1)Test substance identification; (2) substance purity statement; (3) the source/origin information of the substance; (4) information on physicochemical properties of the test item given; (5) cell culture description; (6) the source/origin of cell culture; (7) necessary information on cell culture properties, conditions of cultivation and maintenance; (8) the method of ALA administration; (9) doses or concentration statement; (10) frequency and duration of exposure as well as time-points of observations statement; (11) have negative controls; (12) have positive controls; (13) the number of replicates; (14) are the study endpoint(s) and their method(s) of determination clearly described?; (15) is the description of the study results for all endpoints investigated transparent and complete?; (16) are the statistical methods for data analysis given and applied in a transparent manner?; (17) is the study design chosen appropriate for obtaining the substance-specific data aimed at?; (18) are the quantitative study results reliable? The values of 0 (not satisfied the criteria) or 1 (satisfied the criteria) point allocated for each item. SG, sequence generation; BC, baseline characteristics; AC, allocation concealment; RH, random housing; BI, blinding of investigators; ROA, random outcome assessment; BOA, blinding of outcome assessor; IOD, incomplete outcome data; SOR, selective outcome reporting.
